# Dietary Pattern and Its Association with the Prevalence of Obesity and Related Cardiometabolic Risk Factors among Chinese Children

**DOI:** 10.1371/journal.pone.0043183

**Published:** 2012-08-14

**Authors:** Xianwen Shang, Yanping Li, Ailing Liu, Qian Zhang, Xiaoqi Hu, Songming Du, Jun Ma, Guifa Xu, Ying Li, Hongwei Guo, Lin Du, Guansheng Ma

**Affiliations:** 1 National Institute for Nutrition and Food Safety, Chinese Center for Disease Control and Prevention, Beijing, Beijing, China; 2 China Oxford Centre for International Health Research, Cardiovascular Institute & Fu Wai Hospital, Chinese Academy of Medical Sciences & Peking Union Medical College, Beijing, Beijing, China; 3 Health Science Center, Beijing University, Beijing, Beijing, China; 4 Shandong University, Jinan, Shandong, China; 5 Public Health College, Haerbin Medical University, Haerbin, Heilongjiang, China; 6 Fudan University, Shanghai, Shanghai, China; 7 Guangzhou Center for Diseases Prevention and Control, Guangzhou, Guangdong, China; Tulane School of Public Health and Tropical Medicine, United States of America

## Abstract

**Background:**

The association of dietary pattern with chronic diseases has been investigated widely in western countries. However, information is quite limited among children in China. Our study is aimed to identify the dietary patterns of Chinese children and examine their association with obesity and related cardiometabolic risk factors.

**Methods:**

A total of 5267 children were selected using multistage random sampling from 30 primary schools of 5 provincial capital cities in China. Dietary intake was derived from 24 hour dietary recall for three consecutive days. Anthropometric measurements, glucose and lipid profiles were obtained. Factor analysis combined with cluster analysis was used for identifying major dietary patterns. The associations of dietary patterns with obesity and related cardiometabolic risk factors were examined by logistic regression analysis.

**Results:**

Three mutually exclusive dietary patterns were identified, which were labeled as the healthy dietary pattern, the transitive dietary pattern, and the Western dietary pattern. Compared with children of the healthy dietary pattern, the multiple-adjusted odds ratios (95% confidence interval (CI)) of obesity were 1.11 (0.89–1.38) for children with the transitive dietary pattern and 1.80 (1.15–2.81) for children with the Western dietary pattern, which was 1.31 (95%CI 1.09–1.56) and 1.71 (95%CI: 1.13–2.56), respectively, for abdominal obesity. The Western dietary pattern was associated with significantly higher concentrations of low-density lipoprotein cholesterol (*P*<.001), triglycerides (*P*<.001), systolic blood pressure (*P* = 0.0435) and fasting glucose (*P* = 0.0082) and a lower concentration of high-density lipoprotein cholesterol (*P* = 0.0023), as compared with the healthy dietary pattern.

**Conclusions:**

The Western dietary pattern characterized by red meat, eggs, refined grain and products, was positively associated with odds of obesity, the levels of plasma glucose, low-density lipoprotein cholesterol and triglycerides, and was inversely associated with the level of high-density lipoprotein cholesterol.

## Introduction

As an approach to examine diet-diseases relationship, the analysis of dietary patterns has been implicated frequently since it was introduced in 1980s [1]–[4]. Dietary patterns represent a broader picture of food and nutrient consumption, and may thus be more predictive of diseases risk than individual food or nutrient [4]. It was revealed that distinct dietary patterns were influenced by socio-economic and lifestyle characteristics [5], [6]. China has achieved remarkable economic progress in recent years. Accompanied with these rapid economic changes, dietary pattern is shifting from the traditional pattern with high intake of cereals and vegetables and low intake of animal food, to the Western pattern with high intake of animal foods and other high-energy-dense foods [7], [8].

Following the dietary transition in China, obesity and cardiovascular diseases are accelerating [9]. There are 155 million overweight or obese children around the world, while 12 million of them living in China [10]. With the remarkably increasing in prevalence of obesity among children [11], it has become a public health concern in China [12]. Obesity in children may elevate the risk of cardiovascular diseases [13]–[15] and then predict later health risks [16], [17]. The increase in obesity and obesity-related diseases among children will lead to a significant growth in economic costs [18].

Therefore, it is important to examine the dietary pattern and its association with non-communicable diseases (NCDs) among people in China, especially among children, who are more prone to be influenced by the environments [19]. Dietary pattern and its relationship with diseases were widely investigated in western countries [20], [21]. It was indicated that eating a diet high in vegetables and fiber, and low in meat and fat was associated with a lower risk of obesity and related cardiovascular diseases [22]–[27]. Studies also pointed out that prudent or Mediterranean diet could serve as a healthy dietary pattern to prevent cardiovascular diseases in adults [6], [28], [29]. In China, several dietary patterns had been featured that were associated with different components of metabolic syndrome among adults. He et al. found that the New Affluent/Western dietary patterns were positively associated with glucose tolerance abnormalities (including type 2 diabetes, impaired glucose tolerance, and impaired fasting glucose) [30]; Shanghai Men’s Health Study derived a fruit and milk pattern that the highest score of the fruit and milk pattern was associated with the lowest prevalence of hypertension (22.5% VS. 30.3%, highest vs. lowest quintile of the fruit and milk score) [31]; Shanghai Women’s Health Study identified a dietary pattern low in staple foods and high in dairy milk, which was associated with lower risk of type 2 diabetes [32]. However, dietary pattern and its association with obesity and related cardiometabolic risk factors have not been studied in Chinese children by now.

The present study was aimed to identify the dietary patterns among children; and then to explore whether those dietary patterns are associated with obesity and related cardiometabolic risk factors among children in China.

## Methods

### Subjects

The multistage random cluster sampling procedure was applied to recruit the participants. Beijing and four provincial capital cities including Haerbin, Jinan, Shanghai, and Guangzhou, were selected. Two urban communities from each selected city were randomly selected and three primary schools were then randomly selected from each selected communities. Finally, two classes from each grade of each selected school were randomly selected and all the students in the selected classes were recruited as the study participants. Subjects were recruited and data collection occurred in the summer of 2009.

Totally, we handled out 7500 dietary records in the five cities. Among them, 246 children declined to participate in the study, giving a response rate of 97%. Participants with dietary energy intake less than 350 or greater than or equal to 3500 kcal/day (n = 47), with missing data on gender, age or family environmental factors (n = 752), or missing data of anthropometric measurements or serum profiles (n = 1198) were excluded. Consequently, data from 5267 participants (boys 2643, girls 2624) aged 6–13 years were included in the analysis. No significant differences in age (*P* = 0.1526), sex (*P* = 0.8074) father’s educational level (*P* = 0.2723), and family monthly income per person (*P* = 0.6172) were found between the children who were and who were not included in the present analysis. Differences in mother’ educational level existed between included and excluded children (*P* = 0.0106).

### Socioeconomic Status

The general information including birth weight, feeding types within 4 months after birth, average monthly household income per capita and education level of the parents were collected using a self-administered questionnaire for the parents.

### Physical Activity

Information on physical activity was collected using a validated physical activity questionnaire. The average energy expenditure and duration of total physical activity per day were calculated from the questionnaire [33].

### Assessment of Dietary Intake

Food intake was collected using the 24 hour dietary recall (24 hr-recall) method for three consecutive days (two weekdays and one weekend day). Interviews were conducted by trained investigators. During the interview, samples of local household dishes and utensils (different sizes of bowls, plates, and spoons) were displayed to the children. They were then shown pictures of common foods eaten in these dishes/utensils to indicate portion sizes consumed.

We grouped the foods to 28 groups as below: rice, wheat, refined grains (such as white breads, pasta, muffins, pancakes, and granola bar made by further processing grain powder), other cereals, fried wheat, beans, soybean products, starch tubers, nuts, deep color vegetables, light color vegetables, edible fungi and algae (such as mushrooms, agaric, and kelp), pickled vegetables (such as pickled mustard root, pickled sweet garlic, pickled cucumber and pickled radish), fruits, pork, poultry, seafood, beef/lamb/other red meat, organ meat, processed meat (such as ham, beef jerky and luncheon meat), eggs, milk and yogurt, milk powder and cheese, catsup, beverages, fast-food, sugar, and cakes. Deep color vegetables was classified as carotene content ≥500 µg/100 g and light color vegetables with carotene <500 µg/100 g. The average amount of each kind of food group consumed was calculated by each individual (per day per person).

### Physical Measurements

All the measurements were taken by the trained investigators following the standardized operation procedures. Staff members from Chinese Center for Disease Control and Prevention had trained the investigators from the cooperation center for five days. Fieldwork manuals with guidelines for the conduct of each measurement were provided to every one of the investigators, who had been trained for the standardized operation procedures of each measurement.

Height was measured to the nearest 0.1 cm with a freestanding stadiometer mounted on a rigid tripod (GMCS-I, Xindong Huateng Sports Equipment Co. Ltd., Beijing, China).

Fasting body weight was measured to the nearest 0.1 kg using a balance-beam scale (RGT-140, Weighing Apparatus Co. Ltd. Changzhou Wujin, China) with participants wearing lightweight clothing.

The parents’ height and weight were self-reported by the parents.

BMI was calculated as weight in kilogram divided by the square of height in meter (BMI  =  weight (kg)/(height (meter))^2^).

Waist circumference (WC) was measured midway between the lowest rib and the superior border of the iliac crest with an inelastic measuring tape at the end of normal expiration to the nearest 0.1 cm. If the variation between these two measurements was greater than 2.0 cm, a third measurement was taken and the mean was calculated by using the two closest measurements.

Blood pressure was measured in the seated position using a mercury sphygmomanometer (XJ300/40-1, Made in Shanghai) by trained nurses with at least 10 minutes rest before the measurement. The first and the fifth Korotkoff sounds were used to represent the systolic blood pressure (SBP) and diastolic blood pressure (DBP). Three measurements were taken for all participants to the nearest 2 mmHg, and the average of the last two measurements was used. The mean of the two measurements was calculated as the SBP/DBP.

### Glucose and Lipid Profiles

Fasting venous blood samples (5 mL) were obtained from each participant in the morning after 10–14 hours of overnight fasting. Serum glucose was determined by the glucose-oxidize method (Daiichi Pharmaceutical Co., Ltd, Tokyo, Japan) within 4 hours after the fasting blood sample was obtained. Conventional enzymatic assays were used to measure serum triglycerides (TG), total cholesterol (TC), high-density lipoprotein cholesterol (HDL-C) levels, and low-density lipoprotein cholesterol (LDL-C) levels with 7080 Automatic Analyzer (Daiichi Pharmaceutical Co., Ltd, Tokyo, Japan).

### Quality Control

The same equipments were employed in the five study sites and all the measurements were conducted following standardized operation procedures by the trained investigators. The duplicate measurements in subgroups showed high reproducibility (correlation coefficients of duplicate measurements were 0.99 for height and 0.98 for weight). The intra-observer technical error of measurement (TEM) for waist circumference measurement of our study was 0.3162, relative TEM was 0.5414 and intra-observer reliability (R%) was 99.88%. Every tenth serum sample was measured twice (all correlation coefficients of duplicate measurements 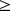
0.98 for glucose and lipid profiles).

### Definitions of Obesity and Related Cardiometabolic Risk

The BMI z-score, based on age and gender, was calculated for each student using WHO growth reference and obesity was defined as BMI z-score>2 SD [34]. Abdominal obesity was defined as age- and sex-specific WC
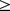
90th percentile proposed by Ji et al. [35].

Obesity-related cardiovascular disease in the present study included high blood pressure, elevated glucose level, hypertriglyceridemia, hypercholesterolemia, dyslipidemia, and metabolic syndrome (MetS).

High blood pressure was defined as SBP and/or DBP>95th age- and sex-specific percentile developed by Mi et al. [36]. Hypercholesterolemia was defined as TG
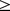
1.7 mmol/L [36]. Hypercholesterolemia was defined as TC
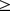
5.18 mmol/L [36]. Dyslipidemia was defined as having one or more of the following three indexes: TG
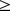
1.7 mmol/L, TC
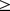
5.18 mmol/L, and HDL-C
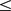
1.04 mmol/L [37]. An elevated glucose level was defined as fasting serum glucose
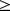
5.5 mmol/L [37]. MetS was defined as obesity
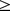
90th percentile assessed by waist circumference and having two or more of the following four indexes: TG
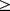
1.7 mmol/L, HDL-C
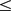
1.04 mmol/L, high blood pressure and fasting serum glucose
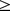
5.5 mmol/L [38].

### Statistical Analysis

In order to identify the major factors accounting for main variance, exploratory factor analysis [39] using the FACTOR procedure was applied. Components with an Eigen value>1, scree test and the interpretability of the factors were considered in determining the number of factors to retain. Four factors with Eigen values>1.20, which together accounted for 24.6% of the total variation, were extracted on the basis of the scree plot and evaluation of the factor loading matrix after orthogonal (varimax) rotation. Food groups with absolute factor loadings ≥0.20 were considered as significantly contributing to the pattern [40]. A high factor score for a given pattern indicated high intake of the foods constituting that food factor and a low score indicated low intake of those foods. A cluster analysis was then performed with the four factor scores of the subjects (calculated from the factor analysis), using the FASTCLUS procedure [41]. This procedure groups subjects into clusters based on Euclidean distances between observations by using K-mean method, which will guarantee the distances between observations in the same cluster are less than all distances between observations in different clusters. The subjects were grouped into three no-overlapping clusters reflecting the dietary patterns.

Children’ gender, birth weight, feeding pattern within 4 months after birth, and parents’ weight, parents’ educational level, and family income level were compared among different dietary patterns using chi-square test. General linear regression models (GLM) were used to compare the difference in weight, height, BMI, WC, cardiometabolic traits, and dietary intake per day among different dietary patterns, after adjustment for the fixed effects, such as sex, age, birth weight, feeding pattern within 4 months after birth, and parents’ weight, parents’ educational level and family income level. Logistic regression random-effect model was used to explore the risk of developing obesity and obesity-related cardiometabolic risk among different dietary patterns after adjustment for the fixed effects and random effects. Cities (study centers) and schools as smaller clusters of observations within cities are treated as two-level random effects. It was considered significant if the *P* value < 0.05. All statistical analyses were done with the SAS 9.2 for Windows (SAS Institute Inc, Cary, NC).

### Ethical Approval

The study protocol was approved by the Ethical Review Committee of the National Institute for Nutrition and Food Safety, Chinese Center for Disease Control and Prevention. Written informed consent forms were obtained from the next of kin, carers or guardians of all the study participants.

## Results

### Characteristics of the Subjects

As showed in Table 1, a total of 5267 children (2643 boys and 2624 girls) were included in the analysis. There was no significant difference in age between boys and girls. Boys had significantly higher height (*P* = 0.0011), weight (*P*<.001), BMI (*P*<.001), and waist circumference than girls (*P*<.001).

**Table 1:** Characteristics of the subjects by gender.*

	Boys	Girls	*P* value
Sample size	2643	2624	
Age (yrs.)	9.5±1.2	9.5±1.2	0.8535
Height (cm)	137.5±8.5	136.8±9.1	0.0011
Weight (kg)	33.9±9.5	31.7±8.4	<.001
Body-mass index (kg/m^2^)	17.6±3.4	16.7±2.9	<.001
Waist circumference (cm)	64.1±54.2	58.0±34.1	<.001
Blood pressure (mm Hg)			
Systolic	106.8±65.7	104.8±62.1	0.0037
Diastolic	69.5±68.1	69.9±64.3	0.0034
Glucose (mmol/L)	4.54±0.52	4.42±0.58	<.001
Cholesterol (mmol/L)			
Total	4.09±0.76	4.13±0.85	<.001
HDL	1.50±0.31	1.47±0.30	0.0827
LDL	2.07±0.62	2.14±0.71	<.001
Triglycerides (mmol/L)	0.81±0.49	0.83±0.44	<.001
Total energy (kcal/d)	1225±591	1218±573	0.1157
Protein (g/d)	53.6±27.8	52.8±27.3	0.3769
Fat (g/d)	36.9±24.6	35.8±22.3	<.001
Carbohydrate (g/d)	171.6±92.1	173.3±90.5	0.3632
Fiber (g/d)	6.1±4.6	6.5±4.6	0.7467

* means ± standard deviation (SD).

*P* values for sex differences are based on t tests.

### Dietary Patterns

Four major factors were extracted through factor analysis using 28 food groups as showed in Table 2. Factor 1 had high positive loadings on rice, refined grains, deep color vegetables, pork, sugar, fish and shrimp, beef, lamb and other red meat. Factor 2 was characterized as high positive loadings on milk and yogurt, eggs, fruit, and vegetables, and high negative loadings on sugar and beef, lamb and other red meat. Factor 3 had high positive loadings on beef/lamb/other red meat, wheat, starch tubers, and light color vegetables. Factor 4 showed high positive loadings on organ meat, pork, seafood, processed meat, edible fungi and algae and light vegetables.

**Table 2:** Pattern loadings of the four major factor solutions after oblique rotation.

Food or food group	factor1	factor2	factor3	factor4
Nuts	0.05	0.12	0.01	0.10
Other cereals	0.10	0.07	0.04	0.08
Organ meat	0.01	−0.09	−0.06	**0.66**
Edible fungi andalgae	0.06	0.09	0.06	**0.59**
Processed meat	0.00	0.07	0.03	**0.20**
Pork	**0.44**	0.08	−0.01	**0.31**
Fish and shrimp	**0.33**	0.03	0.01	**0.25**
Rice	**0.74**	−0.02	−0.01	−0.04
Refined grain	**0.73**	−0.09	−0.20	−0.07
Poultry	**0.24**	−0.10	−0.10	0.15
Wheat	**−0.26**	0.13	**0.60**	0.05
Beef/lamb/other red meat	**0.21**	**−0.21**	**0.66**	−0.06
Starch tubers	−0.07	0.14	**0.60**	0.02
Light color vegetables	0.07	**0.20**	**0.30**	**0.37**
Sugar	**0.42**	**−0.24**	0.01	0.05
Deep color vegetables	**0.65**	**0.28**	0.07	0.10
Eggs	**0.24**	**0.59**	0.18	−0.06
Milk and yogurt	−0.19	**0.62**	0.01	−0.01
Fruits	−0.01	**0.57**	0.00	0.09
Beans	−0.13	0.00	0.01	−0.15
Soybean products	−0.04	−0.01	0.04	0.10
Salted vegetables	0.03	−0.01	−0.04	−0.09
Catsup	0.17	0.05	−0.05	−0.19
Beverages	0.00	0.01	0.08	−0.10
Fast-food	−0.04	0.09	−0.02	0.01
Milk powder andcheese	0.02	−0.02	0.00	−0.05
Fried wheat	−0.17	0.02	0.00	−0.01
Cakes	0.01	0.12	−0.04	0.03
Eigen value	2.52	1.91	1.26	1.21
Percentage ofvariance (%)	9.0%	6.8%	4.5%	4.3%

A cluster analysis based on the four factors derived three mutually exclusive clusters that formed 69.9% (n = 3679), 26.5% (n = 1395), and 3.7% (n = 193) of total subjects, respectively. According to the characteristic of food intake, the three patterns were labeled as the healthy dietary pattern, the transitive dietary pattern, and the Western dietary pattern. As showed in Table 3, cluster 1 (n = 3679) with the highest scores for factor 2 was labeled as the healthy dietary pattern. The second cluster (n = 1395) with remarkably high scores for factor 4 was labeled as the transitive dietary pattern. The third cluster (n = 193) with highest scores for factor 1 and factor 3 was labeled as the Western dietary pattern.

**Table 3:** Classification of subjects by cluster analysis using factor score.

	Cluster1:	Cluster 2:	Cluster 3:
	Healthy diet	Transitive diet	Western diet
Factor 1*	0.04±1.04^a^	−0.14±0.85^b^	0.32±1.17^c^
Factor 2*	0.19±0.98^a^	−0.42±0.82^b^	−0.58±1.30^b^
Factor 3*	−0.18±0.68	−0.00±0.75^b^	3.43±1.50^c^
Factor 4*	−0.32±0.47^a^	0.85±1.15^b^	−0.22±0.80^a^

Plus-minus values are means ± SD.

* p<0.05 by analysis variance.

abc Values with different superscripts in the same row were significantly different by Duncan’s multiple range test at p<0.05.

The characteristics of children according to the dietary patterns were showed in Table 4. Children with the Western dietary pattern tended to have higher family socioeconomic status. Children with the transitive dietary pattern (1273±566 kcal) and the Western dietary pattern (1927±644 kcal), had significantly higher total energy intake than those (1194±563 kcal) with the healthy dietary pattern (both *P*<.001), as showed in Table 5.

**Table 4:** Characteristics of the study subjects according to dietary patterns.

		Healthy	Transitive					Western					
		(n = 3679)	(n = 1395)					(n = 193)					
Age (yr.) (mean ±SD)		9.5±1.2	9.5±1.1					9.5±1.1					
Height (cm) (mean ±SD)		136.8±8.7	137.6±8.7					137.4±8.7					
Gender [N (%)]§													
Male		1836(49.9)	693(49.7)					114(59.2)					
Female		1843(50.1)	702(50.3)					79(40.8)					
Feeding types after birth within 4 months [N (%)]													
Breast		2436(68.8)	970(71.3)					132(72.2)					
Artificial feeding		448(12.7)	153(11.2)					19(11.1)					
Mixed feeding		609(17.2)		230(16.9)				30(16.7)					
Birth weight [N (%)]§													
Low		134(3.8)	55(4.0)					6(3.3)					
Normal		3026(85.5)	1153(84.7)					152(82.6)					
High		379(10.7)		154(11.3)				26(14.1)					
Father’s weight status [N (%)]													
Normal		1855(52.4)	689(50.6)					106(57.6)					
Overweight		1264(35.7)	502(36.9)					59(32.1)					
Obesity		420(11.9)		171(12.6)				19(10.3)					
Mother’s weight status [N (%)]													
Normal		1810(56.6)	686(55.6)					98(58.3)					
Overweight		1220(38.1)	474(38.4)					58(34.5)					
Obesity		170(5.3)			74(6.0)			12(7.1)					
Father’s educational level [N (%)]§													
Illiteracy/primary/Junior middle school		1522(43.8)	506(38.1)					66(36.7)					
Senior middle/high school		1110(31.9)	426(32.0)					61(33.9)					
Technical school/college/university or above		844(24.3)	398(29.9)					53(29.4)					
Mother’s educational level [N (%)]§													
Illiteracy/primary/Junior middle school		1764(51.1)				569(42.9)		80(44.6)					
Senior middle/high school		955(27.6)	391(29.5)					51(28.5)					
Technical school/college/university or above		735(21.3)	367(27.7)					49(27.9)					
Family monthly income per person [N (%)]§													
≤750 (RMB)		375(11.0)	130(9.8)					30(16.7)					
751–1500 (RMB)		1153(36.5)	385(29.1)					66(36.7)					
1501–2500 (RMB)		936(27.4)	388(29.3)					37(20.6)					
≥2501 (RMB)		957(28.0)				420(31.7)		47(26.1)					

§ There was significant difference among different patterns using chi-square test.

**Table 5:** Dietary intake per day and Cardiometabolic traits of children by their dietary patterns.

	Healthy		Transitive		Western
	(n = 3679)		(n = 1395)		(n = 193)
**Nutrition intake per day**					
Energy(kcal)*	1194±563^a^		1273±566^b^		1927±644^c^
Carbohydrate (g)*	171.0±88.4^a^	169.9±84.4^a^		294.7±124.0^c^	
Fat (g)*	35.2±22.0^a^		40.3±27.0^b^		44.1±24.2^c^
Protein (g)*	50.1±25.0^a^		59.6±29.0^b^		90.5±32.4^c^
Carbohydrate (%)*	56.0±10.8^a^		52.2±11.8^b^		59.2±12^c^
Fat (%)*	26.4±9.4^a^		28.0±10.5^b^		20.8±9.4^c^
Protein (%)*	17.6±4.1^a^		19.8±5.2^b^		20.0±5.1^b^
**Cardiometabolic traits**					
Weight (kg)*	32.3±9.0^a^		34.0±10.0^b^		33.5±9.5^b^
Body-mass index (kg/m^2^)*	17.1±3.0^a^		17.3±3.1^b^		18.0±3.7^b^
Waist circumference (cm)*	58.2±8.6^a^		58.9±9.0^b^		60.3±10.0^c^
Blood pressure (mm Hg)					
Systolic*	105.5±10.5^a^		105.5±10.6^a^		111.7±11.8^b^
Diastolic*	69.4±7.2^a^		69.6±7.2^a^		74.4±7.0^b^
Glucose (mmol/L)*	4.46±0.49^a^		4.50±0.53^a^		4.53±0.55^b^
Cholesterol (mmol/L)					
Total*	4.13±0.76^a^		4.09±0.74^a^		4.00±0.70^b^
LDL*	2.07±0.64^a^		2.18±0.68^b^		2.15±0.57^b^
HDL*	1.49±0.30^a^		1.48±0.29^a^		1.43±0.28^b^
Triglycerides (mmol/L)*	0.91±0.48^a^		0.92±0.56^b^		0.93±0.45^b^

Plus-minus values are means ± SD.

* There are significant difference among different patterns using GLM with p value <0.05, after adjustment for gender, age, by using general linear model factorial analysis, while, the school in study center was treated as a random effect variable.

abc Values with different superscripts in the same row were significantly different by Duncan’s multiple range test at p<0.05.

### Cardiometabolic Traits According to Dietary Patterns

Children with the transitive dietary pattern and the Western dietary pattern, had a significantly higher level of weight, compared with their counterparts with the healthy dietary pattern. The level of fasting glucose among children with the Western dietary pattern was significantly higher than that among children with the healthy dietary pattern (4.53±0.55 mmol/L vs. 4.46±0.49 mmol/L, *P* = 0.0082). TC was significantly higher in the healthy dietary pattern than that in the western dietary patterns (4.13±0.76 mmol/L vs. 4.00±0.70 mmol/L, *P* = 0.0065). Children with the Western dietary pattern had significantly higher LDL-C level (2.15±0.57 mmol/L vs. 2.07±0.64**mmol/L, *P* = 0.0023) but lower HDL-C level (1.43±0.28 mmol/L vs. 1.49±0.30 mmol/L, *P*<.001), compared to their counterparts with the healthy dietary pattern. Children with the transitive dietary pattern and the Western dietary pattern, showed significantly higher TG level than their counterparts with the healthy dietary pattern (the transitive dietary pattern vs. the healthy dietary pattern: 0.92±0.56 mmol/L vs. 0.91±0.48 mmol/L, *P*<.001; the Western dietary pattern vs. the healthy dietary pattern: 0.93±0.45 mmol/L vs. 0.91±0.48 mmol/L, *P*<.001). SBP/DBP was significantly higher among children with the Western dietary pattern than that among children with the healthy dietary pattern (SBP: 111.7±11.8 mm Hg vs. 105.5±10.5 mm Hg, *P* = 0.04 and DBP: 74.4±7.0 mm Hg vs. 69.4±7.2 mm Hg, *P* = 0.0435), Table 5.

### Prevalence and Odds Ratio of Obesity and Related Cardiometabolic Risk Factors According to Dietary Patterns

Table 6 reports the prevalence and odds ratio of obesity and related cardiometabolic disorders according to dietary pattern. Obesity was most prevalent among children with the Western dietary pattern (17.1%), followed by the transitive dietary pattern (10.9%) and the healthy dietary pattern (9.2%). Children with the Western dietary pattern had a significantly higher odds of obesity [*OR* (95%CI): 2.04(1.38–3.02), *P*<.001], compared with children who followed the healthy dietary pattern. After adjustment for children’s gender, age, birth weight, total energy intake, feeding types within 4 months after birth, parents’ BMI, parents’ educational level, and family income level, the association between the Western dietary pattern and obesity was attenuated but still significant [*OR* (95%CI): 1.79(1.20–2.67), *P* = 0.0042]. Compared with children who followed the healthy dietary pattern, the full-adjusted *OR* (95% CI) was 1.80 (95%CI: 1.15–2.81, *P* = 0.0099) for children with the Western dietary pattern, after adjustment for confounding factors in Model 3.

**Table 6:** Prevalence and odds ratio (*OR*) of obesity and related cardiometabolic disorders according to dietary patterns.

	Healthy	Transitive	Western (n = 193)
	(n = 3679)	(n = 1395)	
**Obesity**			
Crude prevalence (%)	9.2	10.9	17.1
Model 1: *OR*(95%CI), *P value*	1	1.21(0.98–1.48)**,** 0.0706	**2.04(1.38–3.02), <.001**
Model 2: *OR*(95%CI), *P value*	1	1.17(0.96–1.44)**,** 0.1284	**1.79(1.20–2.67), 0.0042**
Model 3: *OR*(95%CI), *P value*	1	1.11(0.89–1.38)**,** 0.3501	**1.80(1.15–2.81), 0.0099**
**Abdominal obesity**			
Crude prevalence (%)	13.1	17.3	20.7
Model 1: *OR*(95%CI), *P value*	1	**1.39(1.17–1.64), 0.0078**	**1.74(1.21–2.49), <.001**
Model 2: *OR*(95%CI), *P value*	1	**1.35(1.14–1.60), 0.0214**	**1.64(1.14–2.35), 0.0016**
Model 3: *OR*(95%CI), *P value*	1	**1.31(1.09–1.56), 0.0374**	**1.71(1.13–2.56), 0.0093**
**High blood pressure**			
Crude prevalence (%)	12.3	11.7	13.7
Model 1: *OR*(95%CI), *P value*	1	0.95(0.78–1.45), 0.5635	1.21(0.84–1.75), 0.5587
Model 2: *OR*(95%CI), *P value*	1	0.93(0.77–1.12), 0.6138	1.14(0.74–1.74), 0.5973
Model 3: *OR*(95%CI), *P value*	1	0.89(0.73–1.08), 0.6483	0.93(0.60–1.46), 0.7636
**Elevated Glucose Level**			
Crude prevalence (%)	2.0	2.1	3.1
Model 1: *OR*(95%CI), *P value*	1	1.02(0.66–1.57), 0.9323	1.56(0.67–3.63), 0.3028
Model 2: *OR*(95%CI), *P value*	1	0.94(0.61–1.46), 0.9637	1.57(0.67–3.67), 0.3129
Model 3: *OR*(95%CI), *P value*	1	0.95(0.61–1.47), 0.9740	1.58(0.66–3.79), 0.3406
**Hypertriglyceridemia**			
Crude prevalence (%)	4.2	4.0	5.2
Model 1: *OR*(95%CI), *P value*	1	0.94(0.69–1.29), 0.7195	1.24(0.64–2.39), 0.5189
Model 2: *OR*(95%CI), *P value*	1	0.95(0.69–1.30), 0.6985	1.25(0.65–2.42), 0.5135
Model 3: *OR*(95%CI), *P value*	1	0.91(0.67–1.25), 0.5608	1.06(0.53–2.09), 0.8738
**Hypercholesterolemia**			
Crude prevalence (%)	9.2	8.0	8.9
Model 1: *OR*(95%CI), *P value*	1	0.87(0.69–1.08), 0.0998	0.97(0.58–1.61), 0.2018
Model 2: *OR*(95%CI), *P value*	1	0.82(0.65–1.02), 0.0835	0.95(0.57–1.59), 0.5314
Model 3: *OR*(95%CI), *P value*	1	0.81(0.65–1.02), 0.0726	0.80(0.47–1.35), 0.3985
**Dyslipidemia**			
Crude prevalence (%)	16.4	15.7	16.8
Model 1: *OR*(95%CI), *P value*	1	0.95(0.80–1.12), 0.8933	1.03(0.70–1.52), 0.5255
Model 2: *OR*(95%CI), *P value*	1	0.93(0.79–1.10), 0.9011	1.01(0.68–1.49), 0.7357
Model 3: *OR*(95%CI), *P value*	1	0.91(0.77–1.08), 0.2755	0.82(0.55–1.23), 0.3305
**MetS**			
Crude prevalence (%)	0.7	1.9	0.9
Model 1: *OR*(95%CI), *P value*	1	2.36(0.93–6.88), 0.4193	1.10(0.38–4.21), 0.7588
Model 2: *OR*(95%CI), *P value*	1	2.32(0.87–6.17), 0.5362	1.08(0.31–3.81), 0.8512
Model 3: *OR*(95%CI), *P value*	1	2.24(0.82–5.92), 0.5869	1.03(0.29–3.96), 0.8917

Model 1: Logistic regression random-effects model adjusted for gender and age;

Model 2: Logistic regression random-effects model adjusted for Model 1 variable and feeding types, birth weight, parents’ weight, parents’ educational level, average family income per month per capita and study center (school in center);

Model 3: Logistic regression random-effects model adjusted for Model 2 variable and total energy intake (kcal/d) and physical activity energy expenditure (quartile).

The prevalence of abdominal obesity among children with the healthy, the transitive and the Western dietary patterns was 13.1%, 17.3% and 20.7%, respectively. Both children with the transitive dietary pattern and children with the Western dietary pattern had significantly higher odds of abdominal obesity. The multiple-adjusted *OR* (95%CI) were 1.35 (1.14–1.60) for the transitive dietary pattern and 1.64 (1.14–2.35) for the Western dietary pattern, as compared with the healthy dietary pattern, after the adjustment of age, sex, family socioeconomic status and life style factors. After further adjustment of dietary energy intake and physical activity energy expenditure, the odds of abdominal obesity attenuated to 1.71 (95%CI: 1.13–2.56, *P* = 0.0093) among children with the Western dietary pattern, and 1.31 (95%CI: 1.09–1.56, *P* = 0.0374) among children with the transitive dietary pattern, as compared to children with the healthy dietary pattern.

There was no significant difference in prevalence of the other cardiometabolic risk factors among children with different dietary patterns.

## Discussion

Factor analysis, as a multivariate method, represents an alternative approach to the evaluation of foods and nutrients consumption since the identification of patterns that allows us to examine the effect of diet as a whole [40]. The factor is the combined index of all the food items. Food items that were more positively correlated with the factor, would contribute more to this factor. Through cluster analysis based on the four factors, three major dietary patterns were identified among Chinese children and labeled as the healthy dietary pattern, the transitive dietary pattern and the Western dietary pattern in the current study. The healthy dietary pattern was associated with the lowest prevalence of total obesity and abdominal obesity, while children who followed the Western dietary pattern had the highest likelihood of total obesity and abdominal obesity. The Western dietary pattern was also positively associated with LDL, TG, SBP and glucose level, and inversely associated with high HDL level.

Our results are comparable to those of previous studies. The third National Health and Nutrition Examination Survey data in US indicated that the prudent pattern was associated with a lower likelihood of obesity (upper tertile vs. lower tertile: OR, 0.62 (95%CI: 0.40–0.96)) [6]; While the Danish cohort found that the prudent pattern was inversely associated with cardiovascular mortality (upper tertile vs. lower tertile: hazard rate ratio, 0.83 (95%CI: 0.69–1.00) for men, 0.55 (0.40–0.77) for women) [42]. The healthy dietary pattern was inversely associated with the likelihood of obesity (OR (95%CI): 0.63(0.40–0.95)) in our study. However, the present study was a cross-sectional study, so we could not rule out the possibility of overestimate of the effect of dietary pattern on risk of obesity. The healthy dietary pattern in present study was similar with the prudent dietary pattern identified in Western countries with high intake of fruits, vegetables and white meat, which were all associated with the low prevalence of obesity and related diseases [43], [44]. Arezoo et al. indicated that a dietary pattern high in meat, soft drinks, sweets, and snacks, might be positively associated with obesity [45]. In our study, children with the Western dietary pattern were more prone to becoming obese; meanwhile, the total energy intake among children with the transitive dietary pattern and the Western dietary pattern was much higher than that in the healthy dietary pattern. Excess energy intake may play a role in the development of obesity [46]. However, the association we observed between the Western dietary pattern and the prevalence of obesity (including abdominal obesity) was still significant, after adjustment for total energy intake and physical activity energy expenditure. A study also showed that maintaining total energy intakes constant, high intake of fruits and vegetables could help to reduce risk of weight gain in persons [47]. Red meat/refined carbohydrates (high in the Western dietary pattern), are positively associated with saturated fatty acids [48], which may lead to insulin resistance and obesity [49]. Vergnaud AC et al. and Patro B et al. indicated that reducing consumption in meat, snacks, beverage, may decrease the risk of obesity [50], [51]. While, the healthy dietary pattern tends to be an abundance of plant-based foods and dairy. Plant-based foods that provide a large quantity of dietary fiber, low energy density, and high water content, may play a vital role in the low prevalence of obesity [52]. And dietary calcium appears to modulate metabolism, with higher calcium diets reducing energy efficiency and instead favoring increased thermogenesis [53]. Except of containing high dietary calcium, dairy is also rich in bioactive compounds that may act with the suppression of 1, 25-dihydroxyvitamin D3 to favorably affect nutrient portioning, metabolic efficiency, and fat loss [54]. Dairy foods as part of a high quality diet afford protective benefits against a rapidly growing list of conditions, including obesity, cancer, and cardiovascular diseases [55]. Our finding is inconsistent with previous studies that cow’s milk consumption was considered as a promoter of most common chronic diseases in Western countries [56], [57]. Whether dairy products affect chronic diseases in different ways among populations with different diary intakes still warrant further studies [58], [59]. Consumption of milk/yogurt, fruits and vegetables plays a vital role in providing a diversified and nutritious diet; together with previous researches, our results suggest that it is beneficial to increase the consumption of milk/yogurt, fruits and vegetables in the prevention of obesity among Chinese children.

The Western dietary pattern was positively associated with high level of glucose, LDL, and SBP, and inversely associated with high HDL level, after adjustment for confounding factors. The Western dietary pattern was also positively associated with a higher mean fasting glucose level (4.53 mmol/L vs. 4.46 mmol/L, P = 0.0082). While, there was no significant difference of the prevalence of elevated glucose level between children with the Western dietary pattern and children with the healthy dietary pattern (3.1% vs. 2.0%, *P* = 0.3406). We did find some trends but did not find significant difference in the likelihood of high blood pressure, hypertriglyceridemia and MetS among children with the Western dietary pattern. Dietary patterns with high consumption of milk/yogurt, fruit and vegetables have been showed to reduce the markers of cardiovascular disease, while, dietary patterns with high red meats and foods rich in refined carbohydrate have been revealed to elevate the risk of cardiovascular diseases [55], [60], [61]. Ample evidence suggests that fruits and vegetables contain a number of bioactive components, including vitamins, sterols, phenolic compounds, and fiber that may protect against various diseases independently [62]. Furthermore, correcting elevations in intracellular calcium results in clinical improvements in blood pressure, insulin resistance, platelet aggregation, and left ventricular hypertrophy [63]. A systematic review study indicated that fruits and vegetables concentrates are effective in significantly improving circulating concentrations of antioxidant vitamins, provitamins, and folate and decreasing markers of oxidative stress [64], which has been associated with a reduced risk of many chronic diseases [65]. Raised blood pressure, impaired glucose tolerance and dyslipidaemia also tended to be clustered in children and adolescents with unhealthy lifestyles and diets, which included a high frequency of eating meat, less physical activities and smoking [66]–[68]. More attention should be paid to the eating behaviors of the children.

Strengthen of the present study was the control of potential confounders in our analysis. Firstly, our design was multiple level cluster sampling, so school in study center was treated as a random effect. Secondly, as genes and feeding types after birth all play a role in the development of obesity [69]–[72], we further adjusted parents’ weight status and children’ birth weight as surrogate of genetic background, and feeding types within 4 months after birth, respectively. Thirdly, we adjusted the physical activity energy expenditure, which played a role in the energy metabolism. We also adjusted the confounding of socioeconomic status in considering that family environment may be associated with both the children’ weight status and their dietary patterns [73], [74].

### Limitations of Study

One limitation of the study was that, we collected the data of food consumption including 3 days in the summer of 2009. Maybe the diet intake reflected the status of the second season of the year, but could not reflect that of the whole year. Secondly, residual confounding effects could not be avoided as an observational study. Thirdly, considering the children’s weak remembrance about foods’ portion size, the 24 hr-recall method for three days may underestimate children’s dietary intake. In order to reduce the potential recall bias, we offered food models and pictures to children to recall the size of the food they consumed. At last, the cross-sectional design disallows a sequence of temporality to be established for dietary patterns and obesity in children. Future prospective cohort studies are warranted to verify our findings.

### Conclusions

The prevalence of obesity and abdominal obesity was relatively lower among children with the healthy dietary pattern, characterized by high intake of milk/yogurt, fruits, eggs and vegetables and low intake of meat such as pork, poultry, organ meat, and beef/lamb/other red meat. The Western dietary pattern, characterized by high intake of red meat, eggs, refined grain and products, was associated with a higher prevalence of obesity and abdominal obesity and the level of plasma glucose, LDL, and TG, and a lower level of plasma HDL cholesterol. In order to prevent obesity and related cardiovascular diseases, we should guide the children to control their consumption of red meat, eggs, cakes, candy and SSBs, and increase the consumption of vegetables, fruit, and fish. Healthy diet behaviors should be fostered from childhood.
